# A Machine-Learning Approach for Estimating Subgroup- and Individual-Level Treatment Effects: An Illustration Using the 65 Trial

**DOI:** 10.1177/0272989X221100717

**Published:** 2022-05-24

**Authors:** Zia Sadique, Richard Grieve, Karla Diaz-Ordaz, Paul Mouncey, Francois Lamontagne, Stephen O’Neill

**Affiliations:** Department of Health Services Research and Policy, London School of Hygiene & Tropical Medicine, London, UK; Department of Health Services Research and Policy, London School of Hygiene & Tropical Medicine, London, UK; Department of Medical Statistics, London School of Hygiene & Tropical Medicine, London, UK; Clinical Trials Unit, Intensive Care National Audit & Research Centre (ICNARC), London, UK; Université de Sherbrooke, Quebec, Canada; Centre de Recherche du Centre Hospitalier Universitaire de Sherbrooke, Quebec, Canada; Department of Health Services Research and Policy, London School of Hygiene & Tropical Medicine, London, UK

**Keywords:** causal forests, heterogeneous treatment effects, machine learning, personalized medicine

## Abstract

Personalizing treatment recommendations or guidelines requires evidence about the heterogeneity of treatment effects (HTE). Machine-learning (ML) approaches can explore HTE by considering many covariates, including complex interactions between them. Causal ML approaches can avoid overfitting, which arises when the same dataset is used to select covariate by treatment interaction terms as to make inferences and reduce reliance on the correct specification of fixed parametric models. We investigate causal forests (CF), a ML method based on modified decision trees that can estimate subgroup- and individual-level treatment effects, without requiring correct prespecification of the effect model. We consider CF alongside parametric approaches for estimating HTE, within the 65 Trial, which evaluates the effect of a permissive hypotension strategy versus usual care on 90-d mortality for critically ill patients aged 65 y or older with vasodilatory hypotension. Here, the CF approach provides similar estimates of treatment effectiveness for prespecified and post hoc subgroups to the parametric approach, and the results of a test for overall HTE show weak evidence of heterogeneity. The CF estimates of individual-level treatment effects, the expected effects of treatment for individuals in subpopulations defined by their covariates, suggest that the permissive hypotension strategy is expected to reduce 90-d mortality for 98.7% of patients but with 95% confidence intervals that include zero for 71.6% of patients. A ML approach is then used to assess the patient characteristics associated with these individual-level effects, and to help target future research that can identify those patient subgroups for whom the intervention is most effective.

**Highlights:**

- This article examines a causal machine-learning approach, causal forests (CF), for exploring the heterogeneity of treatment effects, without prespecifying a specific functional form.
- The CF approach is considered in the reanalysis of the 65 Trial and was found to provide similar estimates of subgroup effects to using a fixed parametric model.
- The CF approach also provides estimates of individual-level treatment effects that suggest that for most patients in the 65 Trial, the intervention is expected to reduce 90-d mortality but with wide levels of statistical uncertainty.
- The study illustrates how individual-level treatment effect estimates can be analyzed to generate hypotheses for further research about those patients who are likely to benefit most from an intervention.

## Introduction

Personalized, stratified, or precision medicine aims to provide the right treatment to the right patients at the right time,^1,2^ which requires reliable evidence on how effectiveness, harms, and costs of alternative treatments differ across patient subgroups, a concept known as heterogeneity of treatment effects (HTEs).^3^ Conceptual frameworks have been proposed for recognizing HTEs,^4–6^ within randomized controlled trials (RCTs)^7,8^ and observational studies,^9,10^ but their implementation tends to rely on fixed parametric models, which raises important methodological challenges.^3^ First, these approaches consider a few “one-at-a-time” prespecified patient subgroups rather than combinations of subgroup variables.^11–14^ Second, fixed parametric models are prone to model misspecification and more flexible models risk “overfitting” to the data at hand. Overfitting can also occur if the same data set is used to select covariate by treatment interaction terms and to make inferences, leading to the estimation of spurious subgroup effects.^15^ While prespecifying subgroups mitigates this risk, it limits what we can learn from the available data at hand.

Causal machine-learning (ML) approaches have the potential to address these problems in estimating HTEs. Athey and Imbens^16^ and Wager and Athey^17^ have extended classification and regression tree (CART) and random forest algorithms to HTE functions. These nonparametric methods can predict HTEs according to observable characteristics by searching over high-dimensional functions of covariates rather than a few prespecified subgroups. A causal tree approach recursively splits the sample to minimize the variability of HTEs within groups defined by the split, and to maximize their variability across groups^16^ but can be inefficient, in the sense that it is not clear which is the best single tree to use. Causal forests (CFs) are ensembles of causal trees and can increase efficiency (reduce variance) by repeatedly estimating causal trees using random subsets of the data, and averaging the predictions to obtain an overall predicted outcome for each individual under each treatment.^17^ These individual-level effects can be aggregated to generate hypotheses for subgroup effects. CFs, like causal trees, avoid overfitting by using honest estimation,^16^ whereby an observation is either used to determine the splits, or to estimate the effects, but not both.

CF has several potential advantages: it incorporates nonlinear relationships between variables, variable selection, uses honest estimation to ensure valid inference, and, unlike some other ML methods (such as random forests), it is specifically designed to estimate causal effects.^18^ An alternative approach to exploring HTE is to apply more flexible “classical” regression models, for instance, by specifying a rich set of interactions between the covariates and the treatment, including splines, and then estimating individual-level treatment effects by contrasting the predicted potential outcomes for each person under each treatment. However, this approach is prone to model misspecification with respect to the selection of interaction terms and how splines are included in the model.^19^ Regularization approaches (e.g., least absolute shrinkage and selection operator [LASSO]^20^) could be used to remove irrelevant interactions, but the subsequent inference must account for this.^21^ Although honest estimation (sample splitting) could be also used for fixed parametric models, whereby part of the data are used in model development and the remaining data are used to fit the model, parametric model specifications are chosen in practice according to within-sample performance (e.g., adjusted *R*^2^ or Akaike information criterion). Moreover, where the number of parameters to be fitted is larger than the number of observations, regularization (e.g., LASSO) would be required.

Recent articles have applied ML approaches to RCTs, recognizing the importance of avoiding overfitting and maintaining valid hypothesis testing.^22^ However, limited research has critically examined ML approaches for estimating subgroup- and individual-level treatment effects in comparative effectiveness studies that intend to inform clinical decision making and raise hypotheses for future research. The aim of this article is to examine a causal ML approach (CF) for estimating subgroup and individual-level effects and contrast it with fixed parametric models as well as to generate hypotheses about subgroups that can be tested in future research. We consider the methods in reanalyzing a multicenter RCT, the 65 Trial.^23^ The article proceeds as follows: in the next section, we introduce the case study, and the section after that provides an overview of the ML methods used, and their implementation in the 65 Trial. The “Discussion” section details how the findings extend the literature and outlines future research priorities.

## Case Study: The 65 Trial

The 65 trial was a pragmatic, multicenter, parallel-group RCT that aimed to assess the effectiveness of reducing vasopressor exposure through permissive hypotension versus usual vasopressor exposure in critically ill patients aged 65 y or older with vasodilatory hypotension.^23,24^ The study recruited patients from 65 National Health Service adult, general, and critical care units across England, Wales, and Northern Ireland who had vasodilatory hypotension. The intervention aimed to reduce the dose and duration of vasopressors by using less restrictive blood pressure targets (mean arterial pressure range 60–65 mm Hg). The primary outcome was 90-d all-cause mortality, with 2463 patients included in the analysis.

The primary publication reported that reducing the exposure to vasopressors through permissive hypotension did not reduce overall 90-d mortality (unadjusted relative risk, 0.93; 95% confidence interval [CI], 0.85 to 1.03; unadjusted absolute difference −2.85; 95% CI,−6.75 to 1.05).^23^ Prespecified, subgroup analyses considered covariates such as age, chronic hypertension, chronic heart failure, atherosclerotic disease, sepsis, receipt of vasopressors at randomization, physiology score, and baseline risk of death (both according to the Intensive Care National Audit & Research Centre [ICNARC] model) and generated further hypotheses for HTEs.^24^ We consider how ML approaches can explore HTEs, and estimate individual-level treatment effects as well as to generate hypotheses (post hoc) for subgroup effects.

## Overview of Methods for Estimating Subgroup- and Individual- Level Treatment Effects

We are interested in estimating the conditional average treatment effects (CATE), that is, the contrast between the 2 treatment arms, conditional on observed baseline covariates X :

(1) $$\tau \left(x\right)=E(Y_{i}\left(1\right)-Y_{i}\left(0\right)|X=x)$$

where $Y_{i}\left(1\right)$ and $Y_{i}\left(0\right)$ are the individual *i*’s potential outcomes with and without treatment, respectively,^25,26^ and X defines the subgroup of interest.

Because it is not possible to observe both potential outcomes simultaneously,^27^ identification assumptions are required to estimate τ(x) from observed data. In an RCT, these assumptions of consistency, no interference and unconfoundedness, or mean exchangeability are plausible, so that, on average, observed and unobserved confounders are balanced between the arms.

One can estimate the overall ATEs using the method of recycled predictions^28^ by first estimating a regression model including an indicator for the group randomized to treatment (D_i_):

$$Y_{i}=X_{i}\beta +\alpha_{1}D_{i}+\epsilon_{i}$$

where $X_{i}$ is a vector of covariates including an intercept, and then calculating the marginal treatment effect by comparing (counterfactual) predictions for every individual under each treatment. The sample average of these effects can be taken over the full sample to obtain the ATE. This approach assumes that the model is correctly specified and can be termed “outcome regression imputation” or “G-computation.”^29^

Studies commonly report CATEs for a defined subgroup rather than across the full range of values *x*, and we refer to this estimand as the group ATE. The indicator $G_{i}$ equals 1 for individuals in the subgroup and 0 otherwise. This definition of subgroups can refer to categorical variables but also to groups defined by thresholds for continuous variables (e.g., according to quintiles). The group ATE is the average effect for individuals for whom $G_{i}$ = 1. Where the interest is in subgroup effects, we can include a main effect for the subgroup indicator ( $G_{i}$ ) and an interaction term between the subgroup and treatment indicators:

$$Y_{i}=X_{i}\beta +\beta_{G}G_{i}+\alpha_{1}D_{i}+\alpha_{2}D_{i}G_{i}+\epsilon_{i}$$

where $\alpha_{G}$ captures difference in the mean outcome between subgroups in the absence of treatment, $\alpha_{1}$ is the treatment effect for those not in $G_{i}$ , and $\alpha_{1}+\alpha_{2}$ is the treatment effect for those in subgroup $G_{i}$ . One could also interact individual coefficients as opposed to a prespecified group indicator; however, such an approach is prone to overfitting.^30^ For a binary outcome, such as mortality, one can use logistic rather than linear regression.

We can obtain the group ATE for subgroup *G* by contrasting predictions for each individual under each treatment level but considering indicators for both the subgroup and the treatment level. Let ${\hat{Y}}_{i}\left(d,I(G=g\right))$ be the predicted outcome under treatment *d* for subgroup level *g* for individual *i*, the interaction effect compares the following 4 predictions for all patients^31^:

$${\hat{Y}}_{i}\left(1,1\right)-\left({\hat{Y}}_{i}\left(0,1\right)+{\hat{Y}}_{i}\left(1,0\right)-{\hat{Y}}_{i}\left(0,0\right)\right)$$

while the total effect for the subgroup can be obtained using

$${\hat{Y}}_{i}\left(1,1\right)-{\hat{Y}}_{i}\left(0,1\right)$$

and the group ATE by taking the sample average of these effects, with standard errors calculated by the nonparametric bootstrap.^32^

If the subgroups are not prespecified, it may be tempting to report subgroup results for those groups with statistically significant and clinically meaningful effects that are potentially due to random chance, rather than “true” HTE.^33^ This problem is compounded if the study reports CIs that do not recognize when subgroups are chosen post hoc from the data at hand.^34^ While prespecifying the subgroups to be considered may mitigate this concern, it may miss subgroups with important effects.

### CF

Athey and Imbens^16^ propose a data-driven approach to estimate individual-level treatment effects that can be aggregated for subgroups of interest while providing valid CIs for treatment effects within prespecified subgroups, with a sample splitting (“honest”) estimation approach. This approach, “causal trees,” has been expanded to CF^17^ and is a nonparametric, tree-based method for estimating HTE that recursively splits the observations into groups, according to whether or not a particular variable exceeds a threshold value, with the variables and thresholds chosen by the algorithm to maximize the variance of the estimated treatment effect, $\hat{\tau }\left(x_{i}\right)$ , for the sample used to define the splits. For instance, the algorithm might initially split the sample into those aged >85 y versus ≤85 y, because this age threshold leads to estimated HTEs that are maximally different between the 2 resulting groups. These groups (leaves) can each be split into further subgroups, possibly using a different variable or the same variable with a different threshold. Thus, subgroups are formed so that the estimated treatment effect is as homogenous as possible within a leaf (created by splitting at the threshold) and as different as possible between leaves. Under unconfoundedness, the mean observed outcome for the individuals under control (treatment), and in the leaf L corresponding to (X=x) can be used to estimate τ(x) within our subgroups at leaf *L* using

$$\begin{array}{c} \hat{\tau }\left(x\right)=\left(\frac{1}{\left|\left\{i:D_{i}=1,X_{i}\in L\right\}\right|}\sum_{\left\{i:D_{i}=1,X_{i}\in L\right\}}Y_{i}\right)\\ -\left(\frac{1}{\left|\left\{i:D_{i}=0,X_{i}\in L\right\}\right|}\sum_{|\left\{i:D_{i}=0,X_{i}\in L\right\}}Y_{i}\right) \end{array}$$

Thus, the estimated effect for the subgroup is the difference in average outcomes for treated versus control units within the leaf of the tree, L , in which the unit lies.

A CF is defined as an ensemble of *B* causal trees, analogous to decision trees and random forests, and implies averaging predictions ${\hat{\tau }}_{b}\left(x\right)$ over a large number of different possible covariate splits to estimate a CATE for each individual in the sample.^17^ The CF aggregates the predictions from the *B* causal trees by averaging them:

$$\hat{\tau }\left(x\right)=\frac{\sum_{b=1}^{B}{\hat{\tau }}_{b}\left(x\right)}{B}$$

The ensemble approach helps reduce variance, smooth sharp decision boundaries as it does not rely on a single set of splits,^17,35^ and yields valid asymptotic CIs for the true underlying treatment effect,^17^ by using sample splitting (“honesty”). An “honest” estimation approach is where each individual response Y_i_ is used either when learning where to split the leaves, or estimating the within-leaf treatment effect, but not both.^17,36^

The CF yields an estimated effect and standard error for each individual by aggregating their estimated effects for the leaves in which they lie, for each tree within the forest. Moreover, CF implements an overall test for treatment effect heterogeneity, the omnibus test, by fitting the individual-level CATEs as a linear function of the out-of-bag CF estimates (For details see Chernozhukov et al), yielding 2 parameters to which we refer here as the ATE and heterogeneity parameters. This allows us to test 1) whether effect estimates are well-calibrated, and 2) whether the CF found heterogeneity. A coefficient of 1 for the ATE parameter indicates that the mean forest prediction is correct, with the associated *P* value interpreted as a test for the null hypothesis of good calibration. Analogously, a coefficient of 1 for the heterogeneity parameter suggests that the heterogeneity estimates from the forest are well calibrated. Thus, if the heterogeneity parameter is positive, the *P* value associated with it can be interpreted as the strength of evidence in favour of the null hypothesis of no heterogeneity, as this provides evidence of a positive association between the estimated heterogeneous treatment effects and the true effects.^36^

CFs can therefore estimate individual HTEs according to complex covariate interactions while being less prone to overfitting than fixed parametric approaches. Following Athey and Wager^36^ and Basu et al,^37^ we estimate a second CF in which, to improve precision we exclude those variables that have a low importance score (below the mean) which indicates that they were not split on often. In low-signal situations, this allows the forest to make more splits on the most important features.^36^ Since an honest estimation approach is again applied, with splits chosen, and effects estimated on separate samples, this step avoids overfitting.

The CF approach is implemented as an adaptive locally weighted estimator.^18^ First, a forest is used to calculate a weighted set of neighbors. The weights are derived from the fraction of trees in the forest in which an observation appears in the same leaf as the unit of interest. Then effects of interest are estimated, applying a plug-in estimating equation to these neighbors. We can then aggregate the individual-level effects to obtain group ATEs, with a variant of doubly robust estimators already implemented in the generalized random forest R package **grf**.^38^ Here we used augmented inverse propensity weighting (AIPW).^36,39^

### Applying Logistic Regression and CF Approaches to Estimate Group ATEs in the 65 Trial

The trial considered the following prespecified subgroups: age (quintiles), chronic hypertension (yes, no), chronic heart failure (yes, no), atherosclerotic disease (yes, no), predicted risk of death (ICNARC prognostic model), Sepsis-3 (no sepsis, sepsis without septic shock, sepsis with septic shock), vasopressors received at randomization (none, norepinephrine <0.1 µg/kg/min, norepinephrine ≥0.1 µg/kg/min, metaraminol, other/combination). We also considered the following additional subgroup variables: sex (male, female), ethnicity (white, Black/Black mixed, Asian/Asian mixed, other/not stated), dependency prior to acute hospital admission (yes/no), mean arterial pressure at randomization (quintiles), source of admission (emergency department [ED]/not in hospital, elective surgery, emergency surgery, other critical care unit, ward or intermediate care area), acute physiology and chronic health evaluation (APACHE) II score (quintiles), ICNARC physiology score (quintiles), cardiopulmonary resuscitation (CPR) within 24 h prior to admission (community CPR, in-hospital CPR, no CPR), and Sequential Organ Failure Assessment (SOFA) score (quintiles). Observations with missing ethnicity data (*n* = 14) were excluded, and for the other baseline covariates, missing data (<0.1% of patients) were handled with multivariate imputation by chained equation.^23^ There were no missing data for the primary outcome.

The estimand of interest was the ATE, defined as a risk difference, or absolute risk reduction (ARR) in 90-d all-cause mortality. For the parametric regression approaches, we used logistic regression as in the primary study.^24^ For each subgroup of interest, we model the log odds of mortality as a function of dummy variables for treatment (randomized arm), a binary subgroup identifier, and treatment by subgroup interaction terms as:

$$log\left(\frac{p_{i}}{1-p_{i}}\right)=\beta_{G}G_{i}+\alpha_{1}D_{i}+\alpha_{2}D_{i}G_{i}+\epsilon_{i}$$

We estimated CATEs for each individual based on their covariate values and subgroup group ATEs, using the CF method for each outcome according to the following steps:

1. We used the tune_causal_forest function in the grf package^38^ to select tuning parameters.^36^
2. We grew an initial CF consisting of 5000 causal trees using the **causal_forest** function in the **grf** package in R,^38^ we ranked those variables included according to their importance in determining splits within these trees, retained those whose importance was above the mean importance, then repeated steps 1 and 2 to obtain the final CF.
3. We used this CF to estimate the CATE for each individual, along with their standard errors, by predicting from the CF using their covariate values.
4. We aggregated these individual CATEs to obtain group ATEs for each subgroup using AIPW.

In step 2 above, the rationale for retaining a subset of the variables is that this enables the forest to make more splits on the most important features in low-signal situations. We assessed the sensitivity of findings to alternative thresholds (0.2 times the mean importance), and to including the full set of variables, and found results were not sensitive to this choice.

We applied the regression and CF approaches to obtain individual- and subgroup-level estimates, from each imputed data set and combined these estimates with Rubin’s formulae to obtain a single set of effect estimates and accompanying measures of uncertainty.^40^ We present the subgroup effects with forest plots. To assess the strength of evidence for HTEs, we applied the omnibus tests for the final estimates of the ARRs.^17^

### Describing the Effect of Covariate Combinations on the Magnitude of the Individual CATEs

To describe covariates associated with larger estimated treatment effects, Nilsson et al.^41^ suggested regressing the estimated individual CATEs on the covariates of interest.

The model for the expected individual treatment effects is then:

$$E\left[ITE|X_{i}\right]=\Delta \alpha +\sum_{j=1}^{J}\Delta \beta_{j}X_{\mathrm{ji}}$$

where Δα represents the treatment effect independent of the covariates (X), that is, the overall treatment effect, and $\Delta \beta_{j}X_{\mathrm{ji}}$ is the effect explained by observable characteristics $X_{\mathrm{ji}}$ .^41^ The Δ indicates that the coefficients represent differences between the 2 potential outcomes underlying the estimated model.

However, this approach assumes linearity and does not allow for interactions between covariates unless prespecified.^i^ This strategy is similar to the meta-learners described by Kunzel et al.,^42^ whereby first-stage models are used to obtain estimates of the individual CATEs. In a second stage, a regression or a supervised ML method is run with the estimated CATEs as a dependent variable in a model on *X*, the effect modifier of interest, thus obtaining an estimate of the group ATE function.

We are interested in exploring the effect of covariate combinations on the CATE estimates, and so we employ a single CART that can recognize interactions between covariates.^ii^ We use the individual-level CATEs estimated by the CF approach as the dependent variable, with the full set of baseline covariates used to determine splits. In theory, we could continue to recursively split the data set until, for binary or categorical variables, all individuals in each leaf have the same outcome. However, this may lead to overfitting and to subgroups that are difficult to interpret. Therefore, we “prune” the tree, by choosing a complexity parameter that imposes a penalty to the tree for having too many splits. Here, we choose a complexity parameter of 0.2, which yields a manageable (≤10) number of subgroups. It should be noted that effects are not homogenous within these subgroups, and further splitting would lead to more precisely estimated effects.

Because a single CART may overfit the data, we conducted 2 further analyses: 1) we applied an honest estimation approach by identifying subgroups on a subset of the data, and then predicting (estimated) effects for these subgroups using the remaining, out-of-sample, data, which provide valid CIs, and 2) we estimated a regression forest and chose the best tree from this forest, that is, the tree that gives predictions that are most representative of the forest’s predictions. To control the depth of this forest, we chose the minimum number of individuals that must be a leaf before further splitting occurs (*minN*). We set *minN* = 1 (giving the deepest possible tree), 50, 100, and 200.

We calculated the proportion of variation (*R*^2^) in estimated CATEs explained by the estimates from each method, in both in-sample and out-of-sample data, with a low *R*^2^ in the out-of-sample data indicating poor performance in explaining the estimated subgroup effects (see Supplementary Table A1). Finally, for the CART and best tree approaches, we calculated the estimated ATEs for the subgroups found using the in-sample data, and report honest estimates in the sense that the out-of-sample data were not used to identify the subgroups.

## Results

### Estimated Group ATEs in the 65 Trial

**Figure 1:**
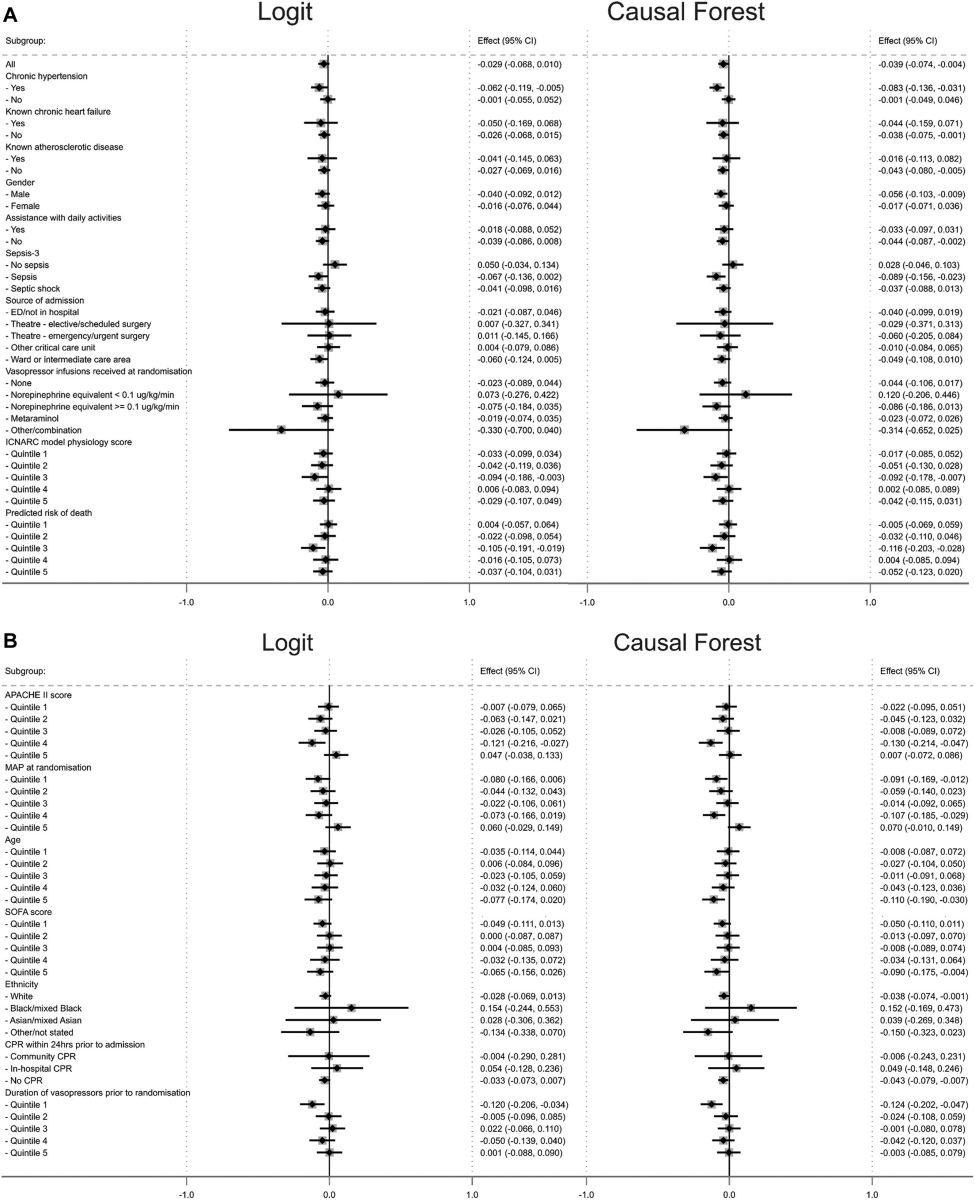
Forest plot of group average treatment effects for 90-d mortality from logistic regression and the causal forest approach.

Baseline characteristics were balanced across the randomized arms (Table 1). The logistic regression model reported an overall ARR for 90-d mortality of −0.029 (SE 0.020), that is 2.9 percentage points (SE 2.0), and the CF approach an overall ARR of 3.9 percentage points (SE 1.8). The forest plots (Figure 1) show that the estimated group ATEs using the logistic regression and CF approaches were similar, with their CIs overlapping. The estimated group ATEs from both methods generated hypotheses that for those patients with chronic hypertension, an ICNARC model physiology score in quintile 3, a predicted risk of death in quintile 3, and APACHE II score in quintile 4, the intervention strategy reduced 90-d mortality. For patients in the oldest quintile, the point estimates for the group ATEs suggested that the permissive hypertension strategy led to reduced 90-d mortality but with 95% CIs that crossed (logistic regression) or were close to zero (CF).

**Table 1:** Baseline Characteristics of All Participants in the 65 Trial

Characteristic	Permissive Hypotension (*n* = 1211)	Usual Care (*n* = 1238)
Age, y, mean (SD)	75.2 (6.9)	75.2 (6.7)
Sex, n (%)		
Male	695 (57.4)	691 (55.8)
Female	516 (42.6)	547 (44.2)
Comorbidities, *n*/*N* (%)		
Chronic hypertension	555/1211 (45.8)	568/1238 (45.9)
Atherosclerotic disease	174/1211 (14.4)	180/1238 (14.5)
Chronic heart failure	134/1211 (11.1)	136/1237 (11.0)
Assistance with daily activities prior to admission, *n* (%)	414 (34.4)	380 (30.9)
Location prior to admission to critical care and urgency of surgery, *n* (%)		
ED/not in hospital	430 (35.5)	419 (33.8)
Theater: elective/scheduled surgery	53 (4.4)	60 (4.9)
Theater: emergency/urgent surgery	256 (21.1)	264 (21.3)
Other critical care unit	14 (1.2)	22 (1.8)
Ward or intermediate care area	458 (37.8)	473 (38.2)
APACHE II score, mean (SD)	20.9 (6.5)	20.6 (6.1)
ICNARC Physiology score, mean (SD)	23.9 (8.8)	23.5 (8.8)
ICNARC^*H-2015*^ predicted risk of death, median (IQR)	0.33 (0.15, 0.60)	0.32 (0.14, 0.61)
Sepsis-3, *n* (%)		
No sepsis	261 (21.6)	275 (22.2)
Sepsis (not in shock)	363 (30.0)	368 (29.7)
Septic shock	587 (48.5)	595 (48.1)
Arterial pressure at randomization (mm Hg), mean (*s*)	69.8 (10.2)	71.0 (11.6)
Vasopressor infusions received at time of randomization, *n* (%)		
None	14 (1.2)	22 (1.8)
Norepinephrine equivalent <0.1 µg/kg/min^d^	140 (11.7)	147 (12.1)
Norepinephrine equivalent ≥0.1 µg/kg/min	645 (54.0)	652 (53.5)
Metaraminol	382 (32.0)	385 (31.6)
Other/combination	14 (1.2)	13 (1.1)
Duration of vasopressor infusion prior to randomization, min, median (IQR)	186 (103, 276)	186 (104, 283)
SOFA score, mean (*s*)	5.5 (1.9)	5.5 (2.0)
Ethnicity, *n* (%)		
White	1,133 (93.6)	1,163 (93.9)
Black/Black mixed	14 (1.2)	12 (1.0)
Asian/Asian mixed	19 (1.6)	18 (1.5)
Other/not stated	45 (3.7)	45 (3.6)
CPR received within 24 h prior to admission, *n* (%)		
Community CPR	26 (2.2)	21 (1.7)
In-hospital CPR	37 (3.1)	37 (3.0)
No CPR	1148 (94.8)	1180 (95.3)

APACHE, Acute Physiology and Chronic Health Evaluation; ED, emergency department; ICNARC, Intensive Care National Audit & Research Centre; IQR, interquartile range; SD, standard deviation.

The omnibus test of HTE from the CF approach indicated weak evidence of heterogeneity (*P* value for a test of the null hypothesis of homogeneous treatment effects = 0.083, the HTE coefficient = 1.210, SE = 0.875). This test also suggests that the HTE estimates were well calibrated (with the *P* value associated with the null hypothesis that the HTE estimates were well calibrated = 0.810) and that the mean forest prediction was correct (*P* value for a test of the null hypothesis that the ATE estimate was well calibrated = 0.995).

### Exploring Heterogeneity in Individual CATEs

The distribution of the estimated individual treatment effects can identify individuals for whom the intervention may be expected to be most effective (or harmful) and to generate further hypotheses for subgroup effects.^44^ As with the parametric approach, if subgroups have not been prespecified, subgroup effects based on the individuals’ estimated CATEs using CFs should be interpreted as exploratory.

**Figure 2:**
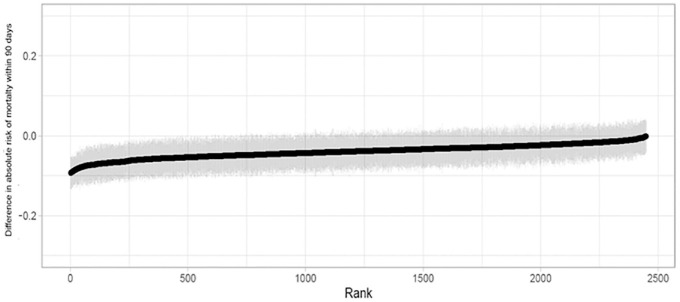
The 95% confidence intervals (light gray) for the estimates of individual-level treatment effects, ordered by the magnitude of the estimates of the individual-level conditional average treatment effects (black line).

The individual-level CATEs for mortality were estimated using the CF method and ranged from −11.0% to 0.8% (Figure 2). These estimated individual-level treatment effects suggest that the permissive hypotension strategy reduces 90-d mortality for 98.7% of patients, but there is great uncertainty with 95% confidence intervals that include zero for the majority (78.2%) of patients. For 28.4% (*n* = 696) of patients, the CATE estimates were negative with CIs below zero, suggesting that for these individuals, we can be relatively certain that the permissive hypertension strategy would be expected to reduce mortality.

### Effect of Covariate Combinations on the Magnitude of the Individual CATEs

**Figure 3:**
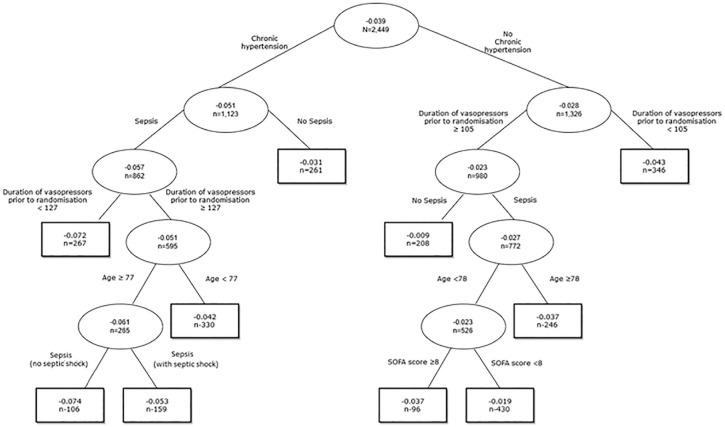
Pruned decision tree for individual-level conditional average treatment effect estimates using causal forest for 90-d mortality.

We find that the pruned CART on the full data set identified 10 subgroups with estimates of individual-level CATEs for 90-d mortality that were sufficiently different to justify splitting, given the choice of complexity parameter (Figure 3). The ARR estimates differed between 5.1 and 2.8 percentage points, for those who had chronic hypertension versus those who did not. The chronic hypertension subgroup was split further into those with sepsis (ARR = 5.7%) and those without (ARR = 3.1%). Within the “no sepsis” subgroup, the heterogeneity was insufficient to justify further splitting, but for the sepsis subgroup, there was considerable heterogeneity when splitting further, according to the duration of vasopressor received prior to randomization, age, and septic shock or not. For patients who did not have chronic hypertension, there was considerable heterogeneity, with further subgroups identified based on a combination of covariates such as duration of vasopressors, sepsis, and SOFA score.

We can interpret these individual-level group ATEs as the expected effect of the permissive hypotension strategy versus usual care, for an individual chosen at random within that subgroup. The findings raise the hypothesis that the permissive hypotension strategy is more effective (ARR = 7.4%) in those subgroups of patients who have chronic hypertension and sepsis, who received vasopressors for at least 128 min before randomization, were aged at least 77 y, and who had not developed septic shock (Figure 3).

Supplementary Table A1 in the appendix compares the performance of ordinary least squares (OLS), CART, and best-tree approaches in terms of the proportion of variation in the HTE explained and the number of subgroups identified. When the maximum depth is used for the best tree, 161 subgroups are identified, which explain 97.5% of the variation in estimated individual-level HTEs. However, when we require at least 200 observations per group, we identify 3 groups that can explain 86.2% of the variation, suggesting that fairly coarse groupings may be beneficial in understanding heterogeneity. By contrast, the OLS model had lower explanatory power (65.6%). Supplementary Tables A2, A3, and A4 report the identified subgroup effects and CIs in sample and out of sample. The CART estimated in an honest fashion identified similar subgroups to the CART estimated on the full sample. The best-tree approach identified subgroups using similar variables (chronic hypertension, sepsis, duration of vasopressor infusion prior to randomization, and age) to those used by the CART, suggesting that, in this example, subgroup selection was not driven by overfitting CART to the in-sample data.

## Discussion

This article examines and applies a causal ML approach, CF, to complement parametric regression models for estimating subgroup effects. The 65 Trial typifies the setting in which an intervention for a broad patient population (critically ill patients aged ≥65 y) has the potential to benefit some patients but harm others. The paper illustrates the relative advantages of CF in avoiding overfitting by calculating CIs using a sample splitting, “honest” estimation approach. The CF approach avoids assuming a particular parametric regression model is correctly specified, and provides an overall assessment of HTE via the omnibus test. Here, the CF approach provides similar estimates of subgroup effects to those from a fixed parametric method, with the omnibus test reporting weak evidence of heterogeneity (*P* = 0.083). The CF approach also provides estimates of the distribution of the individual-level treatment effects and reports that for 98.7% of patients, the intervention is expected to reduce the individual’s 90-d mortality, although the CIs of the estimates include zero in 71.6% of cases. The post hoc analysis of these individual-level effects raises new hypotheses for future research, in proposing more nuanced subgroup combinations that may modify the relative effectiveness of the intervention, but these warrant careful assessment in further research.

This article contributes to methods for exploring HTE in comparative effectiveness research.^42,45–52^ Previous studies have highlighted the advantages of causal ML approaches in avoiding overfitting or type 1 errors, from using the same data to select and interpret covariate by treatment interaction terms, and reducing reliance on correct model specification.^3^ We add to this previous methodological research in illustrating how an advanced ML approach can provide evidence to inform aggregate- and individual-level decision making, but also to help target future research.

This article extends the published analyses of the 65 Trial^23,24^ in finding evidence of heterogeneity, according to one of the prespecified subgroups (hypertension or not). This reanalysis also considered 9 subgroup variables in addition to those defined in the prespecified analysis plan. Although consideration of these additional variables must be regarded as exploratory, and raising rather than testing hypotheses, their use illustrates how CF methods can consider a fuller list of subgroup variables in the exploration of HTE while avoiding reliance on correct specification of a regression model, which is made more challenging when there is an extensive number of covariates. The post hoc analyses of the individual-level treatment effects illustrates how more nuanced hypotheses can be generated. For example, the results raise the hypothesis that the permissive hypotension strategy is effective for critically ill patients aged ≥65 y who have chronic hypertension, and within that subgroup that the intervention is more effective for patients with sepsis. Before these findings can inform personalized medicine, these hypotheses must be tested in external data sets^53–55^ to assess whether the subgroup combinations proposed are replicated.

When using ML methods to explore HTE for the purposes of targeting future research, it is important to recognize the role of more personalized estimates (individual-level CATEs), which are more nuanced toward individual-level decisions, and aggregated groups ATEs, which are more readily interpreted for national guidelines. The expected value of individualized care^5^ provides a framework to consider when providing recommendations according to individual-level CATEs provides sufficient additional value to justify moving away from subgroup recommendations based on group ATEs.

The finding that the group ATE estimates from a fixed parametric approach were similar to those from a causal ML method may reflect some features of the case study, notably small differences in the magnitude of effect across subgroups, and the moderate sample size, typical of many RCTs. However, this does not imply that a simple parametric method will suffice in other settings. Here, the RCT design ensured a reasonable balance on all potential effect modifiers, which reduces the extent to which the estimates are reliant on correct model specification. In observational studies with large baseline covariate imbalances, if the parametric model is misspecified, then the estimates are liable to be biased.^56,57^ The CF approach based on generalized random forests can be helpful as it uses nonparametric estimation of the propensity for treatment and outcome models, incorporates variable selection, and allows for interactions, which are then combined using augmented inverse propensity weighting to obtain doubly robust individual effect estimates.^58^ Related research in observational studies has developed individual-level instrumental variables to consider the problem of confounding, but also heterogeneity according to unobserved factors.^9,59^ However, the current implementation of these individual-level instrumental variable approaches also relies on the correct specification of the statistical model, and a useful extension would be to incorporate causal ML approaches to subgroup selection in this context, analogous to the approach described in this article.

The article has several limitations. First, we have used a single causal ML approach, CF, which is a principled ML approach for estimating subgroup heterogeneity, but other ML methods warrant consideration. Second, the article considers only CF for the primary clinical endpoint. Currently available software would only allow the CF approach to be applied to cost-effectiveness analysis, if defined with a single compositive endpoint, for example, through net-benefit regression. However, this approach would make restrictive assumptions about correct model specification across the underlying endpoints (e.g., mortality, cost, health-related quality of life). Third, the article considers only causal ML in the context of a single RCT. Fourth, the estimates of group ATE and individual-level CATE are intended to generate rather than test hypotheses, as some of the subgroups considered were not prespecified, and allowance was not made for multiple testing.

The study raises questions for further research. Other causal ML methods are available that can be used to estimate HTEs and may have particular appeal in comparative effectiveness research. The squared loss support vector machine (L2-SVM)^60^ uses separate sparsity constraints for the HTE parameters and the covariate parameters. This is likely to be particularly helpful in settings where treatment has a relatively modest effect on outcomes. The X-learner^42^ allows any supervised learning or regression estimators to be used to estimate the CATE and may be preferred to CF for survival outcomes. Our choice of approach was informed by the fact that forest-based methods have been found to perform well across a range of relevant contexts,^61–64^ and software applying these approaches is available in many commonly used packages (e.g., Python and R). Further research could examine ML methods for providing estimates of group ATEs and individual-level CATE for cost-effectiveness analysis, with bivariate ML approaches that use the multivariate random forest method.^65^ Finally, alternative methods to identify subgroups after estimating the CATEs could be explored, such as causal rule ensembles,^66^ which have been shown to perform well when there is overlap between the confounders and effect modifiers.

Identifying the effects of an intervention within subgroups of the population can help target treatments and lead to overall improvements in population health, given resource constraints. Causal ML methods allow for automated variable selection and can easily relax parametric modeling assumptions. In this case study, in which effects were fairly homogeneous across individuals, a parametric approach provided similar estimates of comparative effectiveness to the CF method. Further research into the relative merits of ML versus parametric regression approaches is warranted in alternative settings, such as the evaluation of complex interventions. Here, treatment effects may be modified by combinations of individual and contextual factors, and hence, flexible approaches may provide more useful evidence for decision making.

## Supplemental Material

sj-docx-1-mdm-10.1177_0272989X221100717 – Supplemental material for A Machine-Learning Approach for Estimating Subgroup- and Individual-Level Treatment Effects: An Illustration Using the 65 Trial Click here for additional data file. Supplemental material, sj-docx-1-mdm-10.1177_0272989X221100717 for A Machine-Learning Approach for Estimating Subgroup- and Individual-Level Treatment Effects: An Illustration Using the 65 Trial by Zia Sadique, Richard Grieve, Karla Diaz-Ordaz, Paul Mouncey, Francois Lamontagne and Stephen O’Neill in Medical Decision Making

## Notes

i. If the individual-level treatment effects (ITE) were quadratic in age, for instance, with positive effects for the young and old and negative effects for middle ages, fitting a linear model without interactions could return a null effect of age even though it may be an important driver of ITEs. In contrast, a tree-based method such as CART can choose to recursively split at different ages, overcoming this limitation.

ii. A comprehensive survey of methods for subgroup identification is provided by Lipkovich et al.^43^
