# Supplementary material for: A Machine-Learning Approach for Estimating Subgroup- and Individual-Level Treatment Effects: An Illustration Using the 65 Trial
Source: Med Decis Making. 2022 May 24;42(7):923–36. doi: 10.1177/0272989X221100717 (PMC9459357; doi:10.1177/0272989X221100717)
Supplement: sj-docx-1-mdm-10.1177_0272989X221100717 – Supplemental material for A Machine-Learning Approach for Estimating Subgroup- and Individual-Level Treatment Effects: An Illustration Using the 65 Trial [file sj-docx-1-mdm-10.1177_0272989X221100717.docx]

**Appendix:**

**Table A1: Comparison of in-sample and out-of-sample predictions of HTEs by subgroup identified using pruned honest CART:**

|  |  |  |  |  |  | **In-sample** |  | **Out-of-sample** |
| --- | --- | --- | --- | --- | --- | --- | --- | --- |
| **Subgroup Defined By:** |  |  |  |  | **Number in Group** | **Effect (95% CI)** | **Number in Group** | **Effect (95% CI)** |
|  |  |  |  |  |  |  |  |  |
| Chronic hypertension | Sepsis (no septic shock) | Age >=75 | - | - | 104 | -0.079 (-0.081, -0.076) | 91 | -0.081 (-0.083, -0.079) |
| Chronic hypertension | Sepsis (no septic shock) | Age <75 | - | - | 71 | -0.054 (-0.056, -0.051) | 93 | -0.052 (-0.054, -0.049) |
| Chronic hypertension | Sepsis (with septic shock) | Duration of vasopressors prior to randomisaton <128 | - | - | 64 | -0.065 (-0.068, -0.062) | 82 | -0.063 (-0.065, -0.06) |
| Chronic hypertension | Sepsis (with septic shock) | Duration of vasopressors prior to randomisaton >=128 | - | - | 186 | -0.045 (-0.047, -0.044) | 171 | -0.044 (-0.046, -0.043) |
| Chronic hypertension | No Sepsis | - | - | - | 123 | -0.031 (-0.033, -0.029) | 138 | -0.032 (-0.034, -0.03) |
| No chronic hypertension | Duration of vasopressors prior to randomisaton <105 | - | - | - | 176 | -0.041 (-0.043, -0.039) | 174 | -0.044 (-0.045, -0.042) |
| No chronic hypertension | Duration of vasopressors prior to randomisaton >=105 | Sepsis | Age >= 78 | - | 121 | -0.037 (-0.039, -0.035) | 125 | -0.036 (-0.038, -0.034) |
| No chronic hypertension | Duration of vasopressors prior to randomisaton >=105 | Sepsis | Age < 78 | SOFA score >= 8 | 49 | -0.038 (-0.041, -0.035) | 46 | -0.037 (-0.04, -0.033) |
| No chronic hypertension | Duration of vasopressors prior to randomisaton >=105 | Sepsis | Age < 78 | SOFA score <8 | 226 | -0.02 (-0.021, -0.019) | 201 | -0.018 (-0.02, -0.017) |
| No chronic hypertension | Duration of vasopressors prior to randomisaton >= 105 | No Sepsis | - | - | 104 | -0.008 (-0.01, -0.006) | 104 | -0.011 (-0.013, -0.008) |

**Table A2: Comparison of in-sample and out-of-sample predictions of HTEs by subgroup identified using the best tree from random forest with minimum depth = 200**

|  |  |  |  |  | **In-sample** |  | **Out-of-sample** |
| --- | --- | --- | --- | --- | --- | --- | --- |
| **Subgroup Defined By:** |  |  |  | **Number in Group** | **Effect (95% CI)** | **Number in Group** | **Effect (95% CI)** |
| No Sepsis | - | - | - | **265** | -0.022 (-0.024, -0.02) | **271** | -0.023 (-0.025, -0.021) |
| Sepsis/Sepsis shock | No chronic hypertension | - | - | **534** | -0.032 (-0.033, -0.031) | **517** | -0.032 (-0.034, -0.031) |
| Sepsis/Sepsis shock | Chronic hypertension | - | - | **425** | -0.058 (-0.059, -0.056) | **437** | -0.057 (-0.059, -0.056) |

**Table A3: Comparison of in-sample and out-of-sample predictions of HTEs by subgroup identified using the best tree from random forest with minimum depth = 100:**

|  |  |  |  |  | **In-sample** |  | **Out-of-sample** |
| --- | --- | --- | --- | --- | --- | --- | --- |
| **Subgroup Defined By:** |  |  |  | **Number in Group** | **Effect (95% CI)** | **Number in Group** | **Effect (95% CI)** |
| Chronic hypertension | No Sepsis | - | - | **123** | -0.031 (-0.033, -0.028) | **138** | -0.032 (-0.034, -0.03) |
| Chronic hypertension | Sepsis/Sepsis shock | Minutes of treatment pre randomisation > 81 | - | **118** | -0.074 (-0.077, -0.072) | **149** | -0.071 (-0.073, -0.069) |
| Chronic hypertension | Sepsis/Sepsis shock | Minutes of treatment pre randomisation <= 81 | - | **307** | -0.051 (-0.053, -0.05) | **288** | -0.050 (-0.052, -0.048) |
| No chronic hypertension | Minutes of treatment pre randomisation > 81 | - | - | **116** | -0.044 (-0.047, -0.042) | **124** | -0.047 (-0.049, -0.044) |
| No chronic hypertension | Minutes of treatment pre randomisation <= 81 | No Sepsis | - | **116** | -0.010 (-0.012, -0.007) | **114** | -0.011 (-0.014, -0.009) |
| No chronic hypertension | Minutes of treatment pre randomisation <= 81 | Sepsis/Sepsis shock | Age <= 77 years | **314** | -0.025 (-0.026, -0.023) | **277** | -0.024 (-0.025, -0.022) |
| No chronic hypertension | Minutes of treatment pre randomisation <= 81 | Sepsis/Sepsis shock | Age > 77 years | **130** | -0.038 (-0.041, -0.036) | **135** | -0.037 (-0.039, -0.035) |

**Table A4: Proportion of variation in individual level CATEs explained by method:**

| Version | Proportion of variation in HTEs explained (R^2^)  **In-sample** | Proportion of variation in HTEs explained (R^2^)  **Out-of -sample** | Number of subgroups identified |
| --- | --- | --- | --- |
| OLS (Nilsson et al) | 71.4% | 65.6% | N/A† |
| Honest CART | 83.6% | 81.9% | 17 |
| Pruned Honest CART | 93.5% | 93.4% | 10 |
| Honest Regression Forest (Min N = 1) | 98.0% | 97.5% | 161 |
| Honest Regression Forest (Min N = 50) | 94.1% | 93.4% | 12 |
| Honest Regression Forest (Min N = 100) | 91.6% | 91.0% | 7 |
| Honest Regression Forest (Min N = 200) | 86.9% | 86.2% | 3 |

† OLS does not identify subgroups.
